# An intersectionality approach to Indigenous oral health inequities; the super-additive impacts of racism and negative life events

**DOI:** 10.1371/journal.pone.0279614

**Published:** 2023-01-23

**Authors:** Lisa Jamieson, Xiangqun Ju, Dandara Haag, Pedro Ribeiro, Gustavo Soares, Joanne Hedges

**Affiliations:** 1 Australian Research Centre for Population Oral Health, The University of Adelaide, Adelaide, SA, Australia; 2 Australian Research Centre for Population Oral Health, Adelaide Health & Medical Sciences Building, The University of Adelaide, Adelaide, SA, Australia; New York City Department of Health and Mental Hygiene, UNITED STATES

## Abstract

**Objectives:**

Indigenous Australians experience cumulative forms of oppression. Using intersectionality as the underlying analytical framework, and with oral health as an outcome, we demonstrate how oppressions are interlinked and cannot be treated in isolation. The study aimed to quantify the cumulative effect of two forms of oppression on Indigenous Australian oral health inequities.

**Methods:**

This observational study was conducted Feb 2018—Jan 2020. Recruitment occurred through Aboriginal Community Controlled Health Organisations in South Australia, Australia. Eligibility included identifying as Indigenous, residing in South Australia and aged 18+ years. Socio-demographic factors, health-related characteristics, experience of racism, negative life events and self-reported oral health outcomes were collected. The main outcomes were fair/poor self-rated oral health and oral health related quality of life, measured by OHIP-14. Effect-measure modification was used to verify differences on effect sizes per strata of negative life events and racism. The presence of modification was indicated by Relative Excess Risk due to Interactions (RERIs).

**Results:**

Data were obtained for 1,011 participants, median age 37 years, 66% female and 63% residing in non-metropolitan locations. Over half (52%) had experienced racism in the past 12 months and 85% had experienced one or more negative life events. Around one-third (34%) rated their oral health as fair/poor and the mean OHIP-14 score was 17. A higher proportion of participants who had experienced both racism and negative life events (46%) were male (52%), aged 37+ years (47%), resided in metropolitan locations (57%), reported difficulty paying a $100 dental bill (47%), had fair/poor self-rated oral health (54%) and higher mean OHIP-14 scores (20). The RERIs observed were 0.31 for fair/poor self-rated oral health and 0.23 for mean OHIP-14. The positive RERIs indicated a super-additive effect between racism, negative life events (effect modifier) and self-reported oral health outcomes.

**Conclusion:**

The more oppressions participants experienced, in the form of racism and negative life events, the greater the burden of poor self-reported oral health. The study is one of the first to use intersectionality as a theory to explain oral health inequities as experienced by Indigenous Australians.

## Introduction

Intersectionality theory was first termed by Crenshaw [1], who argued that race- and gender-based inequities needed to be understood through the lens of multiple marginalisations, such as those African American women experience. She argued that such oppressions were mutually constituted and diminished when analytical approaches, which typically informed social policy, treated gender and race as separate constructs. A multiple approach assumes that multiple oppressions (or privileges) experienced by an individual or population group can be layered [2]. Under the lens of intersectionality, an individual’s experience and their health are more than the sum of their parts. The accumulation of oppressions, which include but are not limited to race, sex, income, social class, education, age, sexuality, height, religion and ableism, may be better understood through intersectionality approaches, which use categorisations to examine the cumulative impact of multiple social positions on health inequities [3]. By exploring a wider set of intersectional positions, the impacts of marginalisation as well as privilege across varied social positions can be determined. Hancock suggests that, in large societal groupings, there will be few who experience privilege nearly exclusively, without also experiencing some form of marginalisation [4].

Indigenous Australians are those who identify as Aboriginal and/or Torres Strait Islander and have resided in Australia for over 65,000 years [5]. Contemporary Indigenous Australians represent 3.3 percent of the total Australian population [6]. There is a substantial life expectancy gap (approximately 7 to 9 years) between Indigenous and non-Indigenous Australians [6], with chronic diseases including cardiovascular disease, chronic kidney disease and Type 2 diabetes contributing 80% to this inequity [7]. These persisting inequalities intersect with factors such as gender and location. Since European settlement, many Parliament Acts have influenced the decisions and lives of Indigenous Australians, including child removal, marriage and employment. From 1810, Indigenous Australians were forcibly relocated to state-sanctioned mission stations, administered by churches. The missions inculcated Western values, largely through the banning of Indigenous languages and livelihoods, and whilst food and shelter were provided, conditions were often substandard. The Australian government formally commenced assimilation policies in 1937 (now known as ‘The Stolen Generations’), whereby many Indigenous children were forcibly removed from their families and placed in foster homes with white carers or institutions [8]. The consequences continue to have devastating impacts on the economic, educational, social cohesion, physical and mental wellbeing of Indigenous Australians today. Intergenerational policies of cultural annihilation, child removal, land dispossession and institutional racism have contributed to the accumulation of oppressions experienced among Indigenous Australians [9].

Oral health is a reflection, over the lifecourse, of structural inequities resulting from unequal access to dental health services and a maldistribution of material resources [10]. Inequities in oral health persist across all disease states, age groups and countries [11], and are particularly high among Indigenous groups [12]. Prior to the 1980s in Australia, the oral health of Indigenous children was than their non-Indigenous counterparts [13]. In recent decades, however, the trend has reversed, with Indigenous children now having up to five times the prevalence of dental disease than non-Indigenous children [14]. In the 2017–18 National Survey of Adult Oral Health, almost half (49%) the Indigenous population had dental disease compared with less than one-quarter (23%) of non-Aboriginal Australians [15]. Almost four-fifths (79%) of Aboriginal Australians had missing teeth, compared with less than 60% of non-Aboriginal Australians. had missing teeth. Structural, interpersonal and intrapersonal racism plays an important role in maintaining these oral health inequalities. In the Australian context, the mechanisms through which racism impacts Indigenous oral health include structural inequalities derived from colonial practices and institutional racism, intergenerational trauma, and unfair treatment in health care [16, 17]. The specific pathways through which racism impacts Indigenous oral health include: (1) institutional racism, which causes inequities in access to culturally safe oral health care; (2) cultural racism, which has direct impacts on biological health through psychological and physiological pathways (increased inflammation caused by heightened stress responses) and; (3) interpersonal racism, which can weaken crucial provider-patient relationships between oral health personnel and the Indigenous populations they are serving.

Dental care for Australian adults is provided largely through the private sector, with publicly-funded care eligible through means-testing. A large proportion of Indigenous Australians are eligible for publicly-funded dental care. The wait lists in the public dental sector are, however, long, with care usually requiring co-payment and the range of available services being less comprehensive than those in the private setting. Approximately one-third of federally-funded Aboriginal Community Controlled Health Organisations (ACCHOs), from whom most Aboriginal Australians receive health care, provide dental services with an out-of-pocket cost [18]. Data from the 2017–2018 National Survey of Adult Oral Health indicated that, compared with non-Indigenous Australians, a higher prevalence of Indigenous Australians delayed dental care due to cost, avoided eating certain foods because of dental problems, had experienced toothache in the last 12 months, felt uncomfortable about their dental appearance and perceived a need for dental care [15]. Racism is a substantial barrier for Indigenous Australian in accessing dental care, which contribute to dental fear, difficulties in making dental appointments and motivation to travel substantial distances [18].

**Fig 1:**
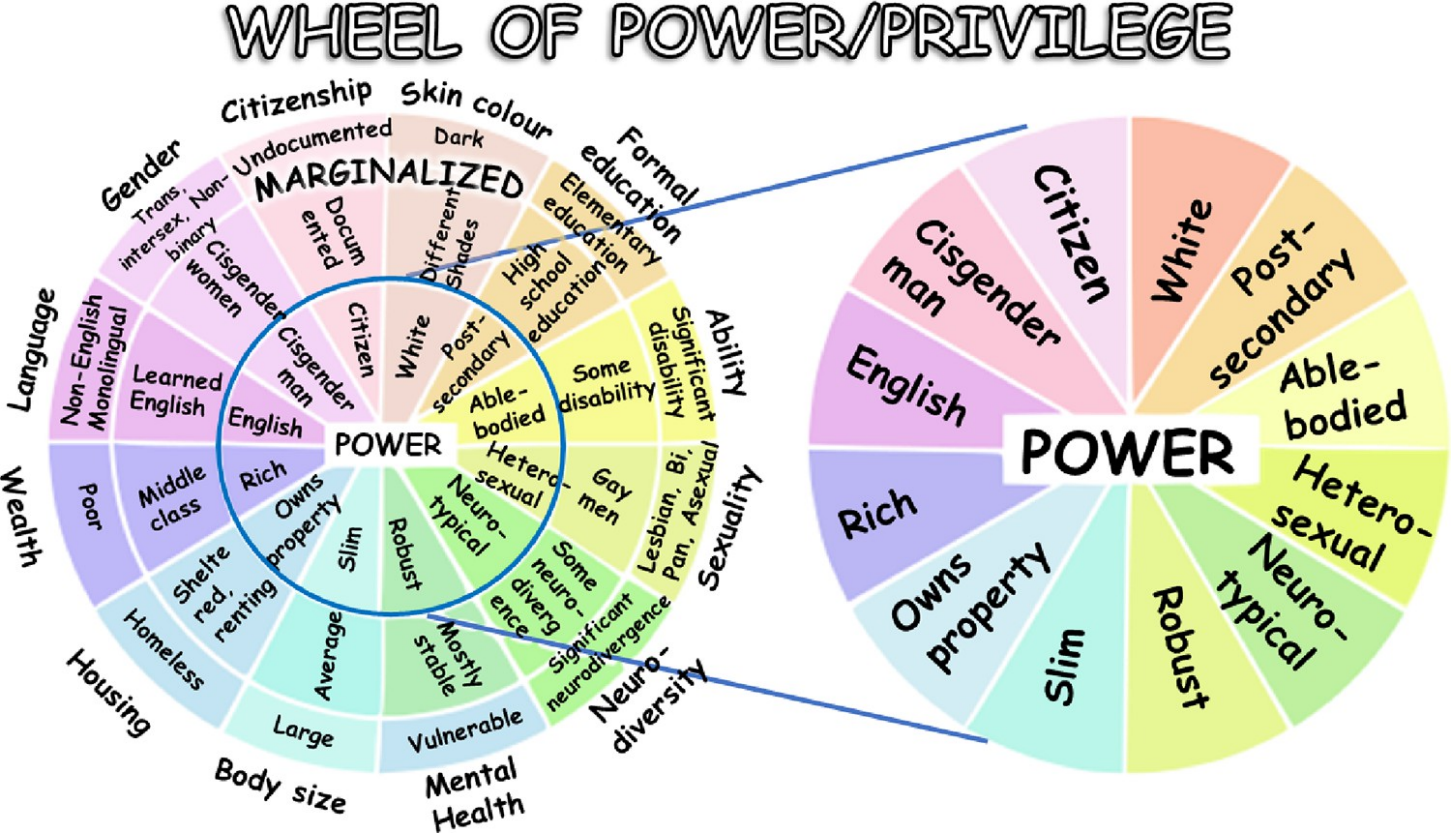
Power and privilege model of intersectionality [2].

It is likely that many Indigenous Australians experience multiple oppressions as represented by the outer margins of Bauer’s ‘Power and Privilege’ model (Fig 1) [19]. The theory of intersectionality suggests that the impact of these multiple oppressions will be greater than the sum of their constituent parts when examining specific health outcomes. Using two self-reported outcomes of oral health, we aimed to quantify, among a large convenience sample of Indigenous South Australians: (1) the prevalence of fair/poor self-rated oral health and oral health impact; (2) the contribution of social, physical and political oppressions on these oral health outcomes and; (3) how an accumulation of oppressions (racism and negative life events) has a super-additive impact on poor oral health. The hypothesis was that, among Indigenous Australians, the effects of racism would be mitigated among those who had experienced a lower magnitude of negative life events in two measures of self-reported oral health.

## Methods

### Study design and participants

We used a large convenience sample (n = 1,011) of adults aged 18+ years who identified as Indigenous in the Australian state of South Australia between Feb 2018 and Jan 2020 as part of a broader study investigating HPV infection and oropharyngeal squamous cell carcinoma [20]. The objectives of the broader study were to examine the prevalence and risk factors for HPV-associated head and neck cancer, and involved collection of self-report and clinical data. Participants were largely recruited through partnering Aboriginal Community Controlled Health Organisations (ACCHOs). The study was governed by an Indigenous Reference Group, with data collected by trained Indigenous research officers. Participants were primarily recruited through ACCHOs, who were key study stakeholders. After having the study explained and signing informed consent forms, participants were requested to complete a questionnaire (with assistance from study staff if required) that contained information on socio-demographic characteristics, health-related factors, experiences of racism, recent history of negative life events and self-reported oral health outcomes.

Ethical approval was received from the University of Adelaide Human Research Ethics Committee (H-2016-246) and the Aboriginal Health Council of South Australia’s Human Research Ethics Committee (04-17-729). Participants gave written, informed consent.

### Outcome variables

Outcomes variables included two self-reported oral health measures; self-rated oral health, a global measure of oral health [21] and the short-form oral health impact profile (OHIP-14) [22]. Self-rated oral health was asked by the question; ‘Would you rate your oral health as’, with response options dichotomised into ‘*fair or poor’* and ‘*good*, *very good or excellent’*. OHIP-14 evaluates seven conceptual dimensions of oral health that affect broader aspects of everyday life. These include functional limitation, physical pain, psychological discomfort, physical disability, psychological disability, social disability and handicap. The leading question was ‘How often in the past year have you’, with five responses (0 = never; 1 = hardly ever; 2 = occasionally; 3 = fairly often; and 4 = very often). Summary scores were obtained, with possible scores ranging from 0 to 56.

### Exposure variable

Experience of racism was assessed using the Measure of Indigenous Racism Experiences (MIRE), which assesses experiences of inter-personal racism across 9 mutually exclusive settings in the last 12 months [23]. The question was: ‘In the last 12 months, have you felt that you have been treated unfairly in any of the following ways because of your racial or ethnic background’? The items evaluate experiences of racism across a range of settings, such as the labour market, housing, health services and education sector. Responses were recorded on a 5-point Likert scale and dichotomized into ‘*strongly disagree*, *disagree*, *neither agree nor disagree’* and ‘*agree and strongly agree’*. A participant was considered to have experienced racism if he/she reported being treated unfairly because of their racial or ethnic background in at least one of the 9 settings.

### Effect modifier

Negative life events were captured using a modified form of the Negative Life Events Scale [24]. The specific question asked ‘In the last 12 months, have you or anyone in your family experienced any of the following: ‘incarceration’, ‘domestic violence’, ‘death’, ‘drug/alcohol misuse’, ‘child removal’, ‘psychological distress’ and ‘cultural or spiritual pain’. Response options were ‘yes’ or ‘no’. A participant was considered to have experienced a recent negative life event if he/she reported one or more adverse events in the last 12 months.

### Covariates

For purposes of this analysis, covariates included age (dichotomised based on a median split for the descriptive analysis), sex, geographic location (‘metropolitan’ or ‘non-metropolitan’) and difficulty paying a $100 dental bill (‘yes’ or ‘no’) as a proxy indicator of socio-economic position.

### Analysis

Basic descriptive analyses were conducted to ascertain frequencies of variables of interest and compared against national population benchmarks where possible. Bivariate and multivariable analyses were then conducted to identify effects between the exposure variable (experience of racism), effect modifier (negative life events) and outcomes (fair/poor self-rated oral health, mean OHIP-14), accounting for other covariates. Effect Measure Modification (EMM) was used to test if the effect between experience of racism (E) and poor self-reported oral health (Y) was stronger among high-negative life event groups (Q). EMM is present when the association between the exposure and the outcome differs across levels of a second exposure (effect modifier). Following the principles of EMM analysis [25, 26], four categories were created representing all possible combinations between the negative life event indicator and experience of racism:

1. No experience of racial discrimination and no adverse life event (PRe0q0);
2. Racial discrimination and no adverse life event (PRe1q0);
3. No racial discrimination and adverse life event (PRe0q1) and;
4. Racial discrimination and adverse life event (PRe1q1).

Prevalence ratios using binomial regression for fair/poor self-rated oral health and mean ratios using generalised linear regressions for mean OHIP-14 for each EMM combination were estimated taking (1) as the reference category. Models included as confounders age, sex, residential location and difficulty paying a $100 dental bill. The Relative Excess Risk due to Interactions (RERIs) were then estimated [25]. RERIs indicate the risk that is in excess of what would be expected if the combination of racial discrimination and negative life event was entirely additive:

$$RERI=PR_{e_{1}q_{1}}-PR_{e_{0}q_{1}}-PR_{e_{1}q_{0}}+PR_{e_{0}q_{0}}$$

A RERI higher than 0 suggests that the effects of the two exposures operating together is higher than that of each added together; a super-additive effect (the effect measure modification is positive). In our analysis, it indicates that the effect of racism interacting with negative life events is higher than the sum of the independent effects of racism and negative life events. A RERI of 0 suggests no effect-measure modification is present, whilst a negative value suggest the effect-measure modification operates in a negative direction [27]. RERIs are interpreted by the direction in which the effect-measure modification occurs, as opposed to RERI size per se [26].

SAS version 9.4 was used for all analyses. Missing data was imputed under the assumption that data was missing at random using the Fully Conditional Specification method with logistic regression for binary variables and linear regression for continuous variables. All missing data were imputed.

## Results

Data from 1,011 Indigenous South Australians aged 18+ years was available. The median age of the sample was 37 years (IQR 27 to 51) and two-thirds (66 percent) were female. Almost two-thirds (63 percent) resided in non-metropolitan locations and three-quarters were financially disadvantaged (difficulty paying $100 dental bill) (Table 1). In the past 12 months, over half the participants (52 percent) had experienced racism in one or more settings and 85 percent had experienced one or more negative life event. The frequencies of responses to the negative life events scale is presented in S1 Appendix. Just over one-third (34 percent) rated their oral health as fair or poor, and the mean OHIP-14 score was 17.0. In comparison with population estimates, a higher proportion of participants in our study were Indigenous, aged 36 years or less, female, resided in non-metropolitan locations, had difficulty paying a $100 dental bill, had experienced racism in the last 12 months, had fair/poor self-rated oral health and higher mean OHIP-14 scores.

**Table 1:** Self-reported oral health, socio-demographic characteristics, health-related characteristics, experience of racism and negative events in last 12 months for Indigenous Australians, compared with national population benchmarks (percent, 95% CI).

	Indigenous study	National population benchmarks (Weighted)*
**Total**	1,011	15,731
***Self-rated oral health***		
Fair or poor	33.5 (30.5–36.4)	23.9 (22.9–24.8)
Good, very good or excellent	63.5 (63.6–69.5)	76.1 (75.2–77.1)
***Mean OHIP-14***	17.1 (16.3–17.9)	5.5 (4.9–6.0)
***Socio-demographic***		
**Age group (years)**		
≥ 37	52.2 (49.1–55.3)	62.1 (61.0–63.2)
< 37	47.8 (44.7–50.9)	37.9 (36.8–39.0)
**Sex**		
Male	33.6 (30.7–36.5)	49.2 (48.1–50.3)
Female	66.4 (63.5–69.3)	50.8 (49.7–51.9)
**Geographic location**		
Non-metropolitan	62.7 (59.7–65.7)	29.1 (28.0–30.3)
Metropolitan	37.3 (34.3–40.3)	70.9 (69.7–72.0)
**Difficulty paying $100 dental bill**		
Yes	75.2 (72.5–77.9)	24.0 (22.9–25.2)**
No	24.8 (22.1–27.5)	76.0 (73.2–76.7)
**Experience of racism in last 12 months (MIRE)**		
Yes	51.8 (48.7–54.9)	11.5 (9.3–13.7)^†^
No	48.2 (45.1–51.3)	88.8 (86.3–90.7)
**Negative life events in last 12 months**		
One or more	84.7 (82.4–86.9)	
None	15.3 (13.1–17.6)	

*2017–18 National Survey of Adult Oral Health [15].

**In NSAOH 2017–18, question was ‘Difficulty paying a $200 dental bill’ [15]

^†^2013 National Dental Telephone Interview Survey [32].

Almost half (46 percent) the participants had, in the last 12 months, experienced both racism and one or more negative life events (Table 2). The proportion who had experienced a negative life event but no racism was 39 percent. Around 6 percent of participants had experienced racism but no negative life event, while 10 percent had experienced no racism and no negative life event. There were stark differences in oral health outcomes depending on the category of oppression used to represent the combined impact of negative life events and experience of racism. For example, the prevalence of fair/poor oral health among those in the category representing the most oppression (both racism and negative life events) was over 7 times that of participants in the category representing the least oppression (no racism and no negative event) (54.3 percent vs 7.7 percent). The same held true for mean OHIP-14; participants who had experienced both racism and one or more negative life events had a 5 point higher mean score than participants experiencing no racism and no negative life event (19.6 vs 14.3).

**Table 2:** Associations between racism, negative life events and poor oral health outcomes, Indigenous South Australian adults, 2018–2020.

	Total sample (n = 1,011)	Experience of racism and negative life event	No experience of racism, negative life event	Experience of racism, no negative life event	No experience of racism, no negative life event
	% (95% CI)	% (95% CI)	% (95% CI)	% (95% CI)	% (95% CI)
**Total**		46.0 (42.9–49.1)	38.7 (35.7–41.7)	5.8 (4.4–7.3)	9.5 (7.7–11.3)
**Age group (years)**					
≥ 37	52.2 (51.5–52.9)	46.6 (42.3–50.9)	38.8 (34.6–43.0)	6.3 (4.2–8.3)	8.3 (6.0–10.7)
< 37	47.8 (47.1–48.5)	45.3 (40.9–49.8)	38.5 (34.2–42.9)	5.4 (3.4–7.4)	10.8 (8.0–13.5)
**Sex**					
Male	33.6 (33.0–34.3)	51.8 (46.4–57.1)	32.1 (27.1–37.2)	7.4 (4.6–10.1)	8.8 (5.8–11.8)
Female	66.4 (65.7–67.0)	43.1 (39.3–46.8)	42.0 (38.3–45.8)	5.1 (3.4–6.7)	9.8 (7.6–12.1)
**Geographic location**					
Non-metropolitan	62.7 (62.1–63.4)	39.7 (35.9–43.5)	44.3 (40.4–48.1)	5.7 (3.9–7.5)	10.4 (8.0–12.8)
Metropolitan	37.3 (36.6–37.9)	56.6 (51.6–61.7)	29.3 (24.6–33.9)	6.1 (3.7–8.5)	8.0 (5.2–10.7)
**Difficulty paying $100 dental bill**					
Yes	75.1 (74.6–75.7)	46.8 (43.3–50.4)	38.9 (35.4–42.4)	5.8 (4.1–7.5)	8.4 (6.5–10.4)
No	24.9 (24.3–25.4)	43.5 (37.4–49.6)	37.9 (32.0–43.9)	5.9 (3.0–8.8)	12.6 (8.5–16.8)
**Self-rated oral health**					
Fair/poor	33.4 (32.7–34.0)	54.3 (49.0–59.6)	32.7 (27.7–37.7)	5.3 (2.9–7.7)	7.7 (4.8–10.5)
Excellent/very good/good	66.6 (66.0–67.3)	41.8 (38.1–45.6)	41.7 (37.9–45.4)	6.1 (4.3–7.9)	10.4 (8.1–12.7)
**OHIP-14 (Mean)**	17.1 (16.3–17.9)	19.6 (18.3–20.8)	14.9 (13.6–16.2)	16.4 (12.8–20.0)	14.3 (11.8–16.7)

In multivariable modelling, after adjusting for covariates, Indigenous Australians who, in the last 12 months, had experienced both racism and one or more negative life events had 1.39 times the prevalence of fair/poor self-rated oral than their counterparts who had experienced no racism and no negative life event (Table 3). The RERI was 0.31 representing a super-additive EMM by negative life event on the additive scale. A similar pattern was observed for mean OHIP-14 (Table 4); after adjusting for covariates, the mean ratio for participants who had experienced racism and negative life events in the last year was 1.35 times higher than the ratio among those who had not experienced either racism or negative life event, with the RERI being 0.23.

**Table 3:** Effect measure modification of negative life event on the effect between experience of racism and fair/poor oral health among Indigenous South Australians.

No negative life event in last 12 months			Negative life event in last 12 months	
Experience of racism	Fair/poor self-rated oral health (yes/no)	Prevalence Ratio (95% CI)	Fair/poor self-rated oral health (yes/no)	Prevalence Ratio (95% CI)
**Never**	26/70	1.0 (Ref)	111/280	1.01 (0.71–1.30)
**At least once**	18/41	1.07 (0.65–1.65)	184/281	1.39 (1.02–1.96)

Relative Excess Risk due to Interaction: 0.31 (95% CI: 0.01–0.66)

Prevalence ratios adjusted for: age, sex, residential location and difficulty paying a $100 dental bill.

**Table 4:** Effect measure modification of negative life event on the effect between experience of racism and mean OHIP-14 among Indigenous South Australians.

No negative life event in last 12 months			Negative life event in last 12 months	
Experience of racism	Mean OHIP-14	Mean ratio	Mean OHIP-14	Mean ratio
	(95% CI)	(95% CI)	(95% CI)	(95% CI)
**Never**	14.3 (11.8–16.7)	1.0 (Ref)	14.9 (13.6–16.2)	1.00 (0.95–1.07)
**At least once**	16.4 (12.8–20.0)	1.12 (1.22–1.30)	19.6 (18.3–20.8)	1.35 (1.27–1.43)

Relative Excess Risk due to Interaction: 0.23 (95% CI:0.06–0.10)

Mean ratios adjusted for: age, sex, education attainment, residential location and difficulty paying a $100 dental bill.

## Discussion

Consistent with our hypothesis, among a large convenience sample of Indigenous Australians, the magnitude of racism effects on two oral health outcomes (poor self-rated oral health, oral health impact) was less among those who had experienced no negative life events. The findings suggest that multiple systems of oppressions have a super-additive impact on health outcomes that need to be accounted for when considering meaningful public health policy. The findings reinforce Crenshaw’s argument that many forms of oppressions intersect and cannot be solved in isolation [1]. It is important to provide context of how negative life events represent or associate with systems of oppression. The best form in which to do so is through the sharing of a narrative that occurred to one study participant (permission obtained). The participant, a middle-aged woman residing in a regional location, had recently lost her mother to illness, had a son incarcerated and was a victim of domestic violence. She lived with a physical disability and had a history of substance misuse. Although she very much valued oral health, she had a very deep fear of the dentist because of past experiences when she was a child. She felt ashamed about the way her teeth looked, which had prevented her from attending a job interview. There were no dental public health services in her town, and she couldn’t afford (and didn’t feel culturally safe) about attending a private clinic, because she couldn’t bring a support person with her and because she believed she would be judged for having poor oral health.

Assessment of health inequities, including oral health inequities, is often done through the lens of one or two categories of difference, which are themselves simplified. For example, socio-economic status is typically assessed using proxies of educational status, occupation and income, but frequently excludes unique intersections between other categories or intersectional positions within a category (recent job loss, single parenthood, mental health issues) [28]. In recent years, population health research has been critiqued for eradicating the context of people’s lives through identifying single sets of health determinants for entire populations [29]. Whilst it could be argued that the findings in this study are also predicated on the simplifying of complex constructs to facilitate data analysis and interpretation, and only two domains of oppressions were considered (racism and negative life events), the unique contribution to the literature is through the use of effect measure modification, and specifically the calculation of RERIs, to quantify the super-additive impacts of multiple oppressions. The impacts, as evidenced, contribute more than the sum of their individual parts; yet policy makers frequently focus on singular oppressions in the form of targeted interventions when distributing scarce health resources.

The prevalence of racism was higher among men, which is corroborated by the findings of Thurber and colleagues who examined experiences of everyday racism among 8100 Indigenous Australians across the nation [30], and may be linked with the higher exposure of men to the types of services known to have high levels of institutional racism, for example, correctional services. The experience of racism was also higher among those residing in urban locations, which is supported by the findings of Markwick et al. among 387 Indigenous adults across the state of Victoria [31]. The more urban a person lives the more contact they have with various sectors of society, which may include higher SES sectors with demonstrated lack of culturally appropriate behaviours. The intersectionality of being male and living in an urban location will have compound effects on the influence of racism on the study outcomes; self-reported oral health and OHIP-14.

In this study, racism (as the exposure) is an intervention point; if participants experienced less racism, the impact of negative life events on self-reported oral health outcomes would have been less. Given that the mechanisms through which racism affects oral health are interconnected, the strategies to mitigate racism in the oral health context must be too. Because most racism impacts arise from structural racism as opposed to inter/intrapersonal racism [32]; we suggest the following recommendations for structural factors that need to change to reduce racism-related impacts on Indigenous oral health inequities: (1) ensuring greater participation of racially marginalized groups with decision-making leadership in oral health care systems, academic institutions and the health policy arena; (2) challenging powerful transnational corporations and their marketing of oral health-damaging foods, beverages and products through specific strategies targeting socially vulnerable groups; (3) having widespread oral health policies that recognize the historical, cumulative impact of racism on socioeconomic factors and the knock-on effect of this on multiple health outcomes; (4) dental services need to be restructured to provide more culturally sensitive care. In the Indigenous Australian context, this means having greater flexible with appointment times, accommodating non-attendance and facilitating the presence of family as support in the clinic; (5) providing greater incentives for oral health personnel to work in communities in which racially underpowered groups reside (and which usually have a low dentist:population ratio); (6) there must be a much stronger emphasis on recruitment and selection of dental students, with adequate representation of racially marginalized groups in dental schools around the globe [33] and; (7) implementing cultural competency training of oral health professionals, including continuing professional development upon graduation.

It is also important to discuss how racism contributes to greater exposure of negative life events. In its inherent nature, experience of racism has psychosocial impacts that directly lead to poor health (accumulation of stress, greater desire for stress-reducing behaviours including tobacco and substance misuse, less desire to keep physically fit and maintain healthy eating habits). The impacts of poor mental and physical health all manifest in outcomes, at a population level, that are reflected in the measure of negative life events; death, incarceration, child removal etc. It is why racism is considered to be such a powerful structural and social determinant of health [34].

The study has a number of strengths and limitations. The main strengths are the excellent engagement with South Australia’s Indigenous communities resulting in strong recruitment and commitment to remain engaged long-term, and use of EMM. The empirical examination of research questions based on intersectionality theory have been greatly facilitated through the field of EMM, which enables simultaneous evaluation of whether the relationship between one exposure varies within strata of another exposure of interest. In our study, we wanted to examine whether the effect between racism and oral health among Indigenous Australians (a group already experiencing oppressions on multiple levels) was modified by levels of negative life events, rather than the combined effect of racism and negative life events per se. To the best of our knowledge, while the modification of the effect between racism and Indigenous oral health has been examined [17, 35], no previous studies have employed EMM analysis to assess whether such effect differs across levels of negative life events; a crucial component in the study of oppressions and intersectionality. Limitations include only two measures of oppression being used and there being no comparator group, for example, non-Indigenous Australians.

In summary, our study is one of first to use intersectionality as a theory to explain Indigenous Australian oral health inequities that explicitly examines simultaneous, additive impacts of racism and negative life events. In support of other intersectionality scholars, we argue that intersectionality cannot be ignored in any research involving, and in partnership with, vulnerable and marginalised groups.

## Supporting information

S1 File (DOCX)

S2 File (DOCX)

S1 Appendix Frequencies of responses to the negative life events scale. (DOCX)

## References

[1] Crenshaw K. Demarginalizing the Intersection of Race and Sex: A Black Feminist Critique of Antidiscrimination Doctrine, Feminist Theory and Antiracist Politics. University of Chicago Legal Forum: 1989. Vol. 1989, Article 8.

[2] Bauer GR. Incorporating intersectionality theory into population health research methodology: challenges and the potential to advance health equity. *Soc Sci Med*. 2014;110:10–7. doi: 10.1016/j.socscimed.2014.03.022 24704889

[3] Samra R, Hankivsky O. Adopting an intersectionality framework to address power and equity in medicine. *Lancet*. 2021;397:857–859 doi: 10.1016/S0140-6736(20)32513-7 33357466 PMC9752210

[4] Hancock AM. When multiplication doesn’t equal quick addition: Examining intersectionality as a research paradigm. *Perspectives on politics*. 2007;5:63–79.

[5] Fahey M, Rossetto M, Ens E, Ford A. Genomic Screening to Identify Food Trees Potentially Dispersed by Precolonial Indigenous Peoples. *Genes (Basel)*. 2022;13:476. doi: 10.3390/genes13030476 35328030 PMC8954434

[6] Australian Institute of Health and Welfare. *Australia’s health 2022*. Canberra: AIHW; 2022.

[7] Australian Institute of Health and Welfare. *Australian Burden of Disease Study*: *impact and causes of illness and death in Aboriginal and Torres Strait Islander people*. Canberra: AIHW; 2016. 10.17061/phrp2741732 29114712

[8] Australian Human Rights Commission. *Face the Facts*: *Questions and Answers about Aboriginal and Torres Strait Islander Peoples*; Australian Human Rights Commission: Canberra, Australia, 2005.

[9] Brown N. History, law, and policy as a foundation for health care delivery for Australian indigenous children. *Pediatr Clin North Am*. 2009;56:1561–76. doi: 10.1016/j.pcl.2009.10.002 19962036

[10] Peres MA, Macpherson LMD, Weyant RJ, Daly B, Venturelli R, Mathur MR, et al. Oral diseases: a global public health challenge. *Lancet*. 2019;394:249–260. doi: 10.1016/S0140-6736(19)31146-8 31327369

[11] Jamieson L, Hedges J, McKinstry S, Koopu P, Venner K. How Neoliberalism Shapes Indigenous Oral Health Inequalities Globally: Examples from Five Countries. *International Journal of Environmental Research and Public Health*. 2020;17:8908 doi: 10.3390/ijerph17238908 33266134 PMC7730877

[12] Tiwari T, Jamieson L, Broughton J, Lawrence HP, Batliner TS, Arantes R, et al. Reducing Indigenous Oral Health Inequalities: A Review from 5 Nations. *J Dent Res*. 2018;97:869–877. doi: 10.1177/0022034518763605 29554440

[13] Barrett MJ, Williamson JJ. Oral health of Australian Aborigines: Survey methods and prevalence of dental caries. *Aus Dent J*. 1972;32:37–50. doi: 10.1111/j.1834-7819.1972.tb02744.x 4402739

[14] Endean C, Roberts-Thomson K, Wooley S. Anangu oral health: the status of the Indigenous population of the Anangu Pitjantjatjara lands. *Aust J Rural Health*. 2004;12:99–103. doi: 10.1111/j.1440-1854.2004.00566.x 15200519

[15] Australian Research Centre for Population Oral Health. *Australia’s Oral Health*: *National Study of Adult Oral Health 2017–18*. Adelaide: The University of Adelaide, South Australia, 2019.

[16] Jamieson L, Peres MA, Guarnizo-Herreño CC, Bastos JL. Racism and oral health inequities; An overview. *EClinicalMedicine*. 2021;34:100827. doi: 10.1016/j.eclinm.2021.100827 33855288 PMC8027540

[17] Hedges J, Haag D, Paradies Y, Jamieson L. Racism and oral health inequities among Indigenous Australians. *Community Dent Health*. 2021;38:150–155. doi: 10.1922/CDH_IADRHedges06 33848408

[18] Australian Medical Association. *AMA Report Card on Indigenous Oral Health*; AMA: Canberra, Australia, 2019

[19] Bauer G. *Quantitative intersectional study design and primary data collection*. Ottawa, CIHR Institute of Gender and Health, Canadian Government. 2021.

[20] Jamieson LM, Garvey G, Hedges J, Leane C, Hill I, Brown A, et al. Cohort profile: indigenous human papillomavirus and oropharyngeal squamous cell carcinoma study—a prospective longitudinal cohort. *BMJ Open*. 2021;11:e046928. doi: 10.1136/bmjopen-2020-046928 34083343 PMC8183277

[21] Locker. What do older adults’ global self-ratings of oral health measure? *J Public Health Dent*. 2005;65:146–52. doi: 10.1111/j.1752-7325.2005.tb02804.x 16171259

[22] Slade. Derivation and validation of a short-form oral health impact profile. *Community Dent Oral Epidemiol*. 1997;25:284–90. doi: 10.1111/j.1600-0528.1997.tb00941.x 9332805

[23] Paradies YC, Cunningham J. Development and validation of the measure of indigenous racism experiences (MIRE). *Int J Equity Health*. 2008;7:9. doi: 10.1186/1475-9276-7-9 18426602 PMC2359753

[24] Kowal E, Gunthorpe W, Bailie RS. Measuring emotional and social wellbeing in Aboriginal and Torres Strait Islander populations: an analysis of a Negative Life Events Scale. *Int J Equity Health*. 2007;6:18. doi: 10.1186/1475-9276-6-18 18001479 PMC2203968

[25] VanderWeele TJ. On the distinction between interaction and effect modification. *Epidemiology*. 2009;20:863–871. doi: 10.1097/EDE.0b013e3181ba333c 19806059

[26] Knol MJ, VanderWeele TJ. Recommendations for presenting analyses of effect modification and interaction. *Int J Epidemiol*. 2012;41:514–520. doi: 10.1093/ije/dyr218 22253321 PMC3324457

[27] Marcelo AK, Yates TM. Young children’s ethnic-racial identity moderates the impact of early discrimination experiences on child behaviour problems. *Cultur Divers Ethnic Minor Psychol*. 2019;25:253–265. 30058832 10.1037/cdp0000220

[28] Layland EK, Maggs JL, Kipke MD, Bray BC. Intersecting racism and homonegativism among sexual minority men of colour: Latent class analysis of multidimensional stigma with subgroup differences in health and socio-structural burdens. *Soc Sci Med*. 2021;293:114602. 34933242 10.1016/j.socscimed.2021.114602 PMC9020748

[29] Raphael D, Bryant T. The limitations of population health as a model for a new public health. *Health Promot Int*. 2002;17:189–99. doi: 10.1093/heapro/17.2.189 11986300

[30] Thurber KA, Colonna E, Jones R, Gee GC, Priest N, Cohen R, et al. Prevalence of Everyday Discrimination and Relation with Wellbeing among Aboriginal and Torres Strait Islander Adults in Australia. *Int J Environ Res Public Health*. 2021;18:6577. doi: 10.3390/ijerph18126577 34207406 PMC8296443

[31] Markwick A, Ansari Z, Clinch D, McNeil J. Experiences of racism among Aboriginal and Torres Strait Islander adults living in the Australian state of Victoria: a cross-sectional population-based study. *BMC Public Health*. 2019;19:309. doi: 10.1186/s12889-019-6614-7 30871531 PMC6419444

[32] Gee GC, Ford CL. Structural racism and health inequities: Old Issues, New Directions. *Du Bois Rev*. 2011;8:115–132. doi: 10.1017/S1742058X11000130 25632292 PMC4306458

[33] Lala R, Baker SR, Muirhead VE. A Critical Analysis of Underrepresentation of Racialised Minorities in the UK Dental Workforce. *Community Dent Health*. 2021;38:142–149. doi: 10.1922/CDH_IADRLala08 33769723

[34] Paradies Y, Ben J, Denson N, Elias A, Priest N, Pieterse A, et al. Racism as a Determinant of Health: A Systematic Review and Meta-Analysis. *PLoS One*. 2015;10:e0138511. doi: 10.1371/journal.pone.0138511 26398658 PMC4580597

[35] Haag D, Schuch H, Ha D, Do L, Jamieson L. Oral Health Inequalities among Indigenous and Non-Indigenous Children. *JDR Clin Trans Res*. 2021;6:317–323. doi: 10.1177/2380084420939040 32731782

