# Supplementary material for: An intersectionality approach to Indigenous oral health inequities; the super-additive impacts of racism and negative life events
Source: PLoS One. 2023 Jan 23;18(1):e0279614. doi: 10.1371/journal.pone.0279614 (PMC9870138; doi:10.1371/journal.pone.0279614)
Supplement: S1 Appendix — (DOCX) [file pone.0279614.s003.docx]

S1 Appendix: Frequencies of responses to the Negative Life Events scale

The section asks questions on important events that might have occurred in your life that impact your health: In the last 12 months please tick if you or anyone else in your family has experienced any following:

|  | % | Cumulative % |
| --- | --- | --- |
| 1: Incarceration | 27.5 |  |
| 2: Domestic violence | 30.4 |  |
| 3: Death | 65.0 |  |
| 4: Drug/alcohol abuse | 42.5 |  |
| 5: Child removal | 17.3 |  |
| 6: Psychological distress (depression/anxiety) | 55.7 |  |
| 7: Cultural/spiritual pain | 33.6 |  |
| 8: Other | 22.9 |  |
|  |  |  |
| No negative life event | 15.3 | 15.3 |
| 1 negative life event | 19.7 | 35.2 |
| 2 negative life events | 15.0 | 50.2 |
| 3 negative life events | 14.1 | 64.3 |
| 4 negative life events | 12.5 | 76.7 |
| 5 negative life events | 7.9 | 84.6 |
| 6 negative life events | 8.6 | 93.2 |
| 7 negative life events | 4.8 | 98.0 |
| 8 negative life events | 2.0 | 100.0 |
